# Hydrogenated monolayer graphene with reversible and tunable wide band gap and its field-effect transistor

**DOI:** 10.1038/ncomms13261

**Published:** 2016-11-10

**Authors:** Jangyup Son, Soogil Lee, Sang Jin Kim, Byung Cheol Park, Han-Koo Lee, Sanghoon Kim, Jae Hoon Kim, Byung Hee Hong, Jongill Hong

**Affiliations:** 1 Department of Materials Science and Engineering, Yonsei University, 50 Yonsei, Seodaemun, Seoul 03722, Korea; 2 Department of Chemistry, Seoul National University, Seoul 08826, Korea; 3 Department of Physics, Yonsei University, Seoul 03722, Korea; 4 Pohang Accelerator Laboratory, Pohang 37673, Korea

## Abstract

Graphene is currently at the forefront of cutting-edge science and technology due to exceptional electronic, optical, mechanical, and thermal properties. However, the absence of a sizeable band gap in graphene has been a major obstacle for application. To open and control a band gap in functionalized graphene, several gapping strategies have been developed. In particular, hydrogen plasma treatment has triggered a great scientific interest, because it has been known to be an efficient way to modify the surface of single-layered graphene and to apply for standard wafer-scale fabrication. Here we show a monolayer chemical-vapour-deposited graphene hydrogenated by indirect hydrogen plasma without structural defect and we demonstrate that a band gap can be tuned as wide as 3.9 eV by varying hydrogen coverage. We also show a hydrogenated graphene field-effect transistor, showing that on/off ratio changes over three orders of magnitude at room temperature.

## Abstract (web summary)

The absence of a band gap in graphene hinders its use in electronics. Here, the authors open a band gap as large as 3.9 electronvolts in graphene grown by chemical vapour deposition by treating it in hydrogen plasma, and then use this material to create a room temperature field- effect transistor.

## Graphical abstract

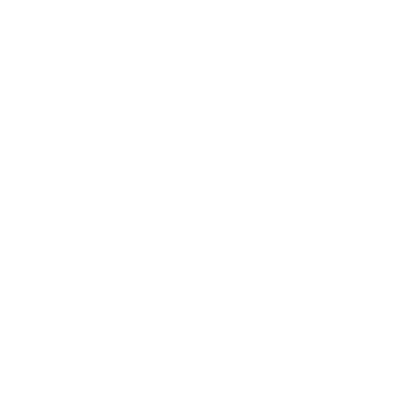

Graphene has opened a new arena in the field of two-dimensional condensed matter physics since its first isolation from graphite by means of micromechanical exfoliation^1^,^2^,^3^. Based on fascinating properties, graphene provides a radically new platform for future electronic and photonic science and technology^4^,^5^,^6^,^7^,^8^. Despite extraordinary physical and chemical properties of graphene, the absence of a sizeable band gap has delayed envisioned graphene-based electronic and photonic science and technology. A number of research groups have taken up the challenge of opening a band gap in graphene and they have mostly followed two main routes: (1) forming graphene nanoribbons^9^,^10^ having a few nanometres in width and (2) a chemical modification of the graphene surface^11^. The graphene nanoribbon has demonstrated its tunability of a magnitude of the band gap and its capability of switchable nano-devices. What makes it challenging is, however, that it is not easy to control the width of ribbons on the order of a nanometre. In contrast, the chemical approach is more practical for large-scale productions and industrial applications. It has also been known to open a band gap of ∼4.0 eV according to theoretical calculations^12^,^13^. Among various chemical approaches, researches on opening the band gap of graphene by hydrogenation have been intensively studied. Not only theoretical works on hydrogenated graphene^12^,^13^ but many experimental efforts on hydrogenation techniques such as a hydrogen-plasma treatment^14^, a reaction with hydrogen atoms in a hydrogen silsesquioxane (HSQ) film^15^,^16^ and an exposure of atomic hydrogen (deuterium) beams^17^ have been reported. In particular, hydrogen-plasma treatment has been regarded as an efficient way to modify the surface of graphene and to apply for a standard wafer-scale fabrication. However, the energetic ions have etched carbon atoms and induced structural damage in graphene. Despite various experimental efforts^9^,^14^,^15^,^16^,^17^,^18^,^19^, none of them has succeeded in observing a sizable band gap of at least ≥1 eV or in managing a device operation yet. The absence of a sizeable band gap in graphene synthesized by the present technologies and experimental challenges originating from the intrinsic nature and extrinsic degradation of graphene are impeding further developments in envisioned carbon-based electronics.

Here, we show that monolayer graphene hydrogenated by indirect hydrogen plasma at room temperature acquires a band gap up to ∼4.0 eV that can be tuned by varying hydrogen coverage while preserving the structural integrity of the original graphene. We first demonstrate that the intrinsic band-gapped graphene we created fully functions as a field-effect transistor (FET), showing that conductivity at gate voltages changes over three orders of magnitude at room temperature.

## Results

### Determination of a band gap by transport measurements

**Figure 1:**
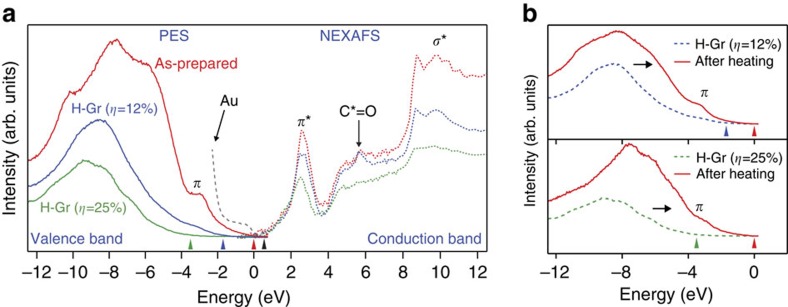
Evolution of the electronic band structure of hydrogenated monolayer graphene (H-Gr) at 300 K. (**a**) PES and NEXAFS spectra of graphene and H-Gr. Red, blue and green arrows indicate the valence-band maximum of as-prepared graphene, H-Gr (*η*=12%) and H-Gr (*η*=25%), respectively. Black arrow means the shift of the conduction band of both H-Gr (*η*=12%) and H-Gr (*η*=25%). The band gap was tunable up to 3.9 eV by the change of hydrogen coverage *η*. (**b**) Recovery of the *π* orbital of graphene in PES spectra after annealing of H-Gr in vacuum at 550 K for 2 h.

Opening a band gap in chemical-vapour-deposited (CVD) graphene^20^,^21^ was possible by exposing pristine graphene to indirect (or remote) hydrogen plasma in vacuum at room temperature. Synchrotron radiation X-ray photoemission spectroscopy (PES) showed that the valence band of hydrogenated graphene was shifted down to energy much lower than that of graphene or Au, as shown in Fig. 1a. (We hereafter refer to hydrogenated monolayer graphene as H-Gr.) The magnitude of the shift increased and then saturated at 3.5 eV (green arrow in Fig. 1) as the exposure time to the hydrogen plasma or hydrogen coverage *η* on graphene increased. We found that the hydrogen coverage *η* saturated at ∼25% when we further increased the exposure time (see Supplementary Fig. 1 and Supplementary Note 1). When the downshift of the valence-band maximum was 1.7 eV (blue arrow in Fig. 1) and 3.5 eV, the *η* for H-Gr was ∼12% and ∼25%, respectively. Small shifts of ∼0.4 eV (black arrow in Fig. 1) were observed in the conduction-band minimum by near-edge X-ray absorption fine structure (NEXAFS) spectroscopy, but these shifts did not change significantly with the coverage. The resulting band gap of CVD graphene was opened as a result of exposure to indirect hydrogen plasma ranging from 2.1 to 3.9 eV.

A close investigation of these spectra reveals that the band-gap opening indeed arises from the hydrogenation of graphene. The *π* and the π* orbitals, a fingerprint of graphene, disappeared and were significantly reduced, respectively, with the coverage. In other words, the *sp*^2^ orbitals rehybridized with the local *sp*^3^ orbitals as a result of hydrogenation. In addition, the C*=O orbital, located at ∼3.2 eV above the π* orbital, also disappeared as the hydrogenation continued, indicating that the hydrogen replaces oxygen chemisorbed on the CVD graphene. It is noteworthy that the pronounced π* band signifies topological ripples and corrugations, and that the intermediate states between π* and σ* contain residual adsorbates commonly observed in CVD graphene^22^. Such defects are extrinsic and are inevitably induced during the fabrication process of CVD graphene^20^,^21^,^23^.

Our *in-situ* heating PES measurements of H-Gr directly prove that our indirect hydrogen plasma indeed inflicts minimal damage on graphene, that is, that H-Gr has no significant defects in the honeycomb basal plane after hydrogenation. (We provide additional evidence for this later.) The indirect plasma technique is well known to significantly reduce damages to a surface, because the surface is not in direct contact with the plasma, which contains energetic particles. Both the band gaps of H-Gr created at an *η* of 12% and 25%, for example, returned to 0 (Fig. 1b) when H-Gr was heated in the PES chamber (<8 × 10^−10^ Torr) at 550 K for 2 h. Besides, the recovery of the *π* orbital below the Fermi energy proves that hydrogen that had bonded with the *p*_z_-orbital of carbon was desorbed during heating and that H-Gr was reduced to metallic graphene. Such reversibility has been observed only when the graphene framework was not seriously damaged by surface treatments. Our observation of the full recovery of H-Gr to graphene bears a critical implication for the modulation of a band gap of graphene: we can control the band gap either by exposing the graphene to indirect hydrogen plasma or by thermal treatment of the resulting H-Gr.

### Optical absorption experiments

**Figure 2:**
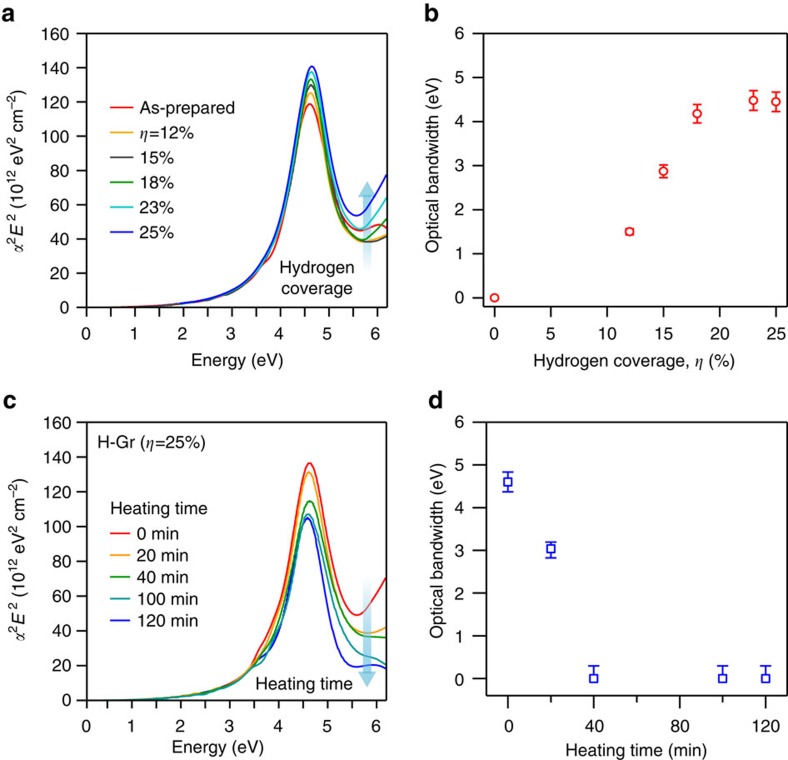
Optical bandwidth and reversibility of H-Gr at 300 K. (**a**,**b**) The evolution of optical (near infrared, visible and ultraviolet) absorption spectra of graphene and H-Gr and their estimated bandwidths as a function of hydrogen coverage *η*. In the figure, *α* is the optical absorption coefficient and *E* is the incident photon energy. The zero optical bandwidth indicates the screening of interband tails by exciton resonance. (**c**,**d**) The full recovery of the optical absorption spectra and bandwidths of H-Gr (*η*=25%) retuning to the pristine graphene state after thermal treatment at 550 K under Ar atmosphere as a function of annealing time. The error bars correspond to ±0.5% uncertainty in the measurement of optical transmission acquired by spectrophotometry.

Not only the transport band-gap measurements (PES and NEXAFS spectroscopy) but also optical absorption experiments support the wide tunability of a band gap of graphene induced by our plasma treatment. Initially, the absorption spectra of graphene acquired from near-infrared to ultraviolet at room temperature in air exhibited a general feature of a *π* plasmon located at ∼4.6 eV due to excitonic Fano resonance^24^. As hydrogenation proceeded, the *π* plasmon peak is blue-shifted by up to ∼0.04 eV (Fig. 2a). The optical bandwidth of H-Gr (as estimated from the higher-lying absorption background) ranged from 1.4 to 4.6 eV, depending on the hydrogen coverage *η* (Supplementary Fig. 2 and Supplementary Note 2), which is different from the bandwidth measured with the synchrotron radiation X-ray source. However, it was also saturated at *η* of 25%. It is not uncommon to see such a difference in the magnitude of the bandwidth estimated by the two measurements: whereas the transport measurement integrates both indirect and direct excitations about the Fermi energy, the optical absorption is mainly due to direct (vertical) band-to-band excitation. Therefore, the optical spectra often reveal the upper bound of the bandwidth in the case of indirect band-gap semiconductors. This is most likely the case in what we observed here—more of which later. Meanwhile, it is worth noting that the crucial reversible recovery from H-Gr to graphene was repeatedly confirmed through *ex-situ* optical absorption experiments. After we annealed H-Gr with an optical bandwidth of 4.6 eV at 550 K for 20 min under Ar atmosphere and carried out optical absorption experiments, the optical bandwidth decreased from 4.6 to 3.0 eV (Fig. 2c,d). The longer thermal treatment accelerated dehydrogenation of H-Gr and restored H-Gr to the pristine state of graphene as indicated by the disappearance of the absorption background.

### Structural integrity

**Figure 3:**
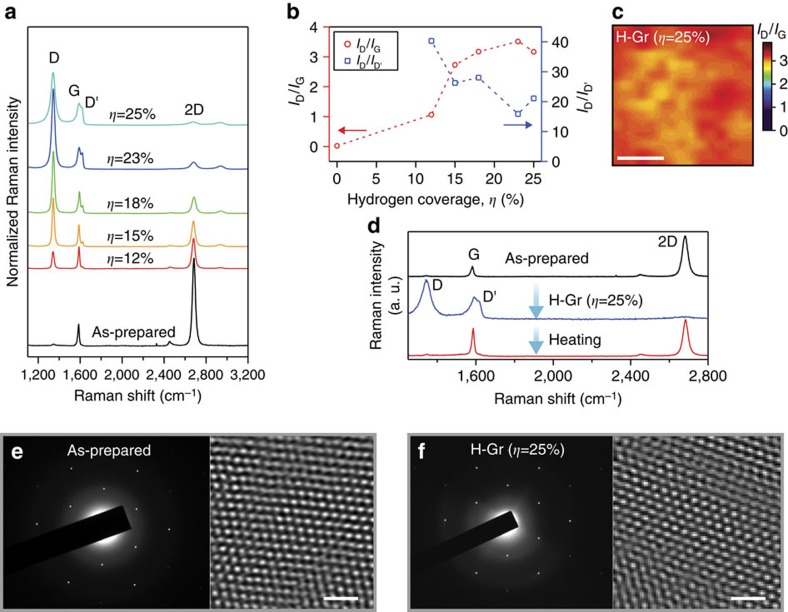
Raman behaviours and atomic images of graphene and H-Gr. (**a**)The Raman changes of H-Gr with an increase in hydrogen coverage *η* at 300 K. (**b**) The ratio of integrated intensity, *I*_D_/*I*_G_ and *I*_D_/*I*_D′_, as a function of hydrogen coverage *η*. (**c**) Raman mapping of *I*_D_/*I*_G_ for H-Gr at *η*=25%. Scale bar, 5 μm. (**d**) The recovery of Raman spectra of H-Gr treated by *ex-situ* heating for the optical absorption experiments. (**e**,**f**) Selected area electron-diffraction patterns and high-resolution transmission electron microscopy images for graphene and H-Gr, respectively. Scale bar, 1 nm.

We found that the hydrogenation or the formation of *sp*^3^ C–H bonds by indirect hydrogen plasma occurred without significant damage to its carbon network. As the hydrogen coverage increased, the Raman spectra evolved in a distinct manner, as shown in Fig. 3a. Although the G and 2D peaks decreased with the coverage, the D and D′ peaks started to appear^25^,^26^,^27^ and then became dominant, which is known to be caused by defects such as *sp*^3^ C–H bonds and/or the broken symmetry of a carbon *sp*^2^ network^14^. Our Raman spectra also reveal two additional features (Fig. 3b). One is that the ratio of the integrated intensity of the D peak to that of the G peak (*I*_D_/*I*_G_) gradually increased^27^ and saturated at ∼3.0 with the coverage of up to 25%. It is noteworthy that the ratio mapping indicated a uniform distribution of the defects throughout the specimen (Fig. 3c). The ratio of the integrated intensity of the D peak to that of the D′ peak (*I*_D_/*I*_D′_) showed a similar saturation tendency and stayed at over 16, never dropping after saturation. The former ratio and its mapping imply that the defects are uniformly distributed and saturated at *η* of 25%, as the distance between defects no longer increases, and the latter ratio shows that the defect is not an atomic-vacancy type but an *sp*^3^ type in H-Gr^28^.

The possibility of broken symmetry of the honeycomb *sp*^2^ network can be ruled out by high-resolution transmission electron microscopy and by the evidence of the return of H-Gr to the pristine graphene state by the aforementioned thermal treatment. Neither structural damage nor disintegration of the innate symmetry of graphene was found in the micrographs (Fig. 3f). The sixfold symmetric dots shown in the electron-diffraction patterns also support the existence of a high-quality honeycomb network for both graphene and H-Gr. The H-Gr treated by *ex-situ* heating for the optical absorption experiments fully recovered the Raman features of graphene (Fig. 3d). The most surprising discovery was that the D peak had completely disappeared after heating. Such Raman behaviours are impossible to observe, unless graphene maintains a two-dimensional carbon network of high quality. Typical damages found in hydrogenated graphene by plasma treatment could be amorphization and void formation, and they render the graphene irreversibly hydrogenated, making it unusable for long-term applications.

### Electrical transport measurements and FET

**Figure 4:**
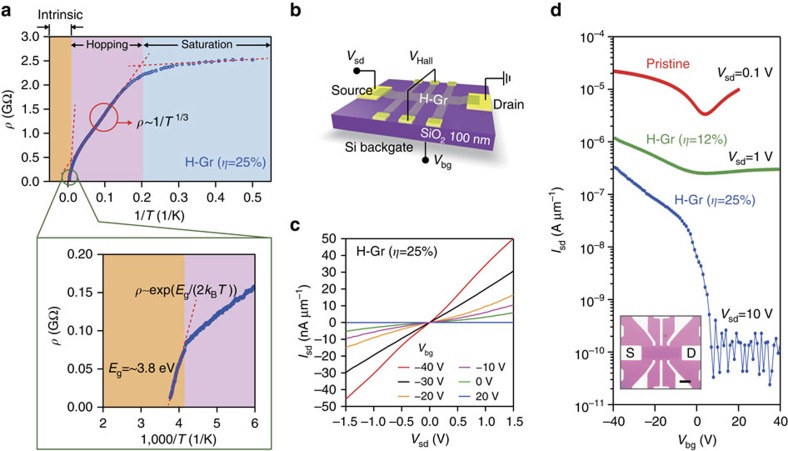
Temperature (*T*)-dependent resistivity (*ρ*) of H-Gr and the characteristics of H-Gr's FETs at 300 K. (**a**) The behaviour of *ρ−*1/*T* of H-Gr (*η*=25%) reveals the prevailing transport mechanism as crystal semiconductors. (**b**) Schematics of H-Gr's FETs and its experimental setup. (**c**) *I*–*V* source-drain characteristics were initially nonlinear but became linear as the gate bias was increased from *−*40 to 20 V. (**d**) Change in current on the sweep of a back-gate voltage (inset: optical image of the devices; scale bar, 10 μm).

The temperature (*T*) dependence of resistivity (*ρ*) of our H-Gr shows that the band gap stems from a long-range order at an *η* of 25%, whereas it can be opened by delocalized defect states at the lower hydrogen coverages (Supplementary Fig. 3 and Supplementary Note 3). As shown in Fig. 4a, the *ρ−*1/*T* of H-Gr (*η*=25%) showed three distinct behaviours: (1) intrinsic excitation, (2) variable-range hopping and (3) saturation towards the Mott minimum conductivity—all of which are essential features observed in crystal semiconductors^29^. It is noteworthy that amorphous or disordered gapped materials such as graphene oxide cannot show such distinct stages^30^. In fact, the band-gap curve-fitted by the relation of *ρ*=*ρ*_0_ exp[*E*_g_/(2*k*_B_*T*)]) in the intrinsic region was ∼3.8 eV, which is close to the band gap (∼3.9 eV) measured by transport band-gap measurements. On the other hand, when hydrogenation coverage was insufficient, the *ρ−*1/*T* showed the behaviour of variable-range hopping conductance, in which *ρ* is proportional to *T*^−1/3^ (not shown here) as others observed^14^.

Judging from the size of a band gap and the temperature-dependent resistivity of our H-Gr showing an *η* of 25%, we have reached the conclusion that our saturated H-Gr can have a form of para-type C_4_H (refs 31, 32, 33), a single-sided hydrogenated graphene. As a matter of fact, hydrogenation in the H-Gr happens most probably on the top surface of graphene transferred onto a SiO_2_/Si substrate, because our hydrogen atoms do not have strong enough energy to penetrate graphene due to the mild and non-destructive nature of indirect hydrogen plasma. According to the electronic band structure calculation based on density functional theory^32^,^33^, C_4_H-type hydrogenated graphene has an indirect band gap of 3.5 eV. This is close to the 3.9 eV that we observed through transport measurements, which provide integrated signals about the Fermi energy. It is worth noting that the density functional theory calculation usually underestimates the value of the band gap. The different magnitudes of the band gaps we measured by X-ray and optical absorption add further weight to our conclusion. The wide bandwidth of ∼4.5 eV estimated by optical absorption is believed to be the result of a direct (vertical) band-to-band excitation: optical absorption has been found to be very sensitive to the direct transitions at high-symmetry points, such as *K*, *M* and *G*, in the Brillouin zone of C_4_H-type hydrogenated graphene. The difference in the magnitude of the measured band gaps may also in part stem from the difference in the degree of hydrogenation or in the distribution of C–H bonds possibly caused by the different substrates used for transport measurements and optical absorption measurements—Si/SiO_2_ and sapphire, respectively (see Methods).

Finally, we demonstrate the functionality of a FET made from H-Gr (Fig. 4b–d). Our proof-of-concept FET made out of hydrogenated monolayer graphene with a band gap of 3.9 eV on 100 nm-thick SiO_2_ gate dielectric showed a dramatic change in current of over 10^3^ on/off ratio on the sweep of a back-gate voltage at room temperature as shown in Fig. 4d. To the best of our knowledge, this is the highest ratio achieved among functionalized graphene-based FETs ever reported and is an additional clear evidence for the intrinsic band gap of our H-Gr being the result of the hybridization of the periodic structure. However, our on/off ratio could not go over 10^4^. We believe such a limit can be ascribed to the high off-current due to leakage current through the thermally oxidized SiO_2_ of poor quality. *I*–*V* source-drain characteristics were initially nonlinear but became linear as the gate bias was increased over –20 V (Fig. 4c, as similarly observed in a wide-band gap FET such as fluorinated graphene FET^34^ and large band gap semiconductor devices^35^. This indicates that the electrical transport process has changed from tunnelling through Schottky contact between metal and H-Gr to resistive conduction due to excitation of electrons into the conduction band of the hydrogenated graphene. When the positive gate bias was increased up to 40 V, the off-current state was maintained at the noise level, that is, it was clearly off-state at gate bias exceeding positive 5 V. Such asymmetric conductance response to the gate bias indicates that our hydrogenated graphene is a *p*-type semiconductor. The increase of the band gap inevitably leads to a decrease in mobility due to the dominant formation of an *sp*^3^ bond^36^. For example, the mobility was ∼4 cm^2^ V^−1^ s^−1^ for the FET with a band gap of 3.9 eV (Supplementary Table 1 and Supplementary Note 4). To show that the mobility can be further increased by improving our primitive proof-of-concept structure and minimizing the substrate effect, we fabricated several other FETs. A top-gated FET with polymethyl methacrylate (PMMA)/Al_2_O_3_ dielectric, for example, exhibited more than double the mobility (∼9 cm^2^ V^−1^ s^−1^), indicating that the performance of FETs can be significantly improved by implementing a high-*κ* gate dielectric and an improved FET structure (Supplementary Fig. 4). We note that our mobility value is significantly lower than the mobility of pristine graphene but is still higher than that of current FETs for flexible electronics, for which graphene is one of the most suitable materials because of its superb mechanical^37^ and optical properties^7^, and large area synthesis technique^20^,^21^. In the case of the FET made out of H-Gr with a band gap of 1.7 eV (*η*∼12%), the on/off ratio was unexpectedly low. This is because the band gap was opened by random hydrogenation^31^, which is evidenced by the temperature-dependent resistivity (Supplementary Fig. 3 and Supplementary Note 3).

The very considerable advantage of our method is that a device can be built entirely of graphene: a conductor, that is, graphene, and a semiconductor and an insulator, that is, our hydrogenated graphene with small and large band gaps^38^. Moreover, H-Gr in this study promise not only to elucidate many controversial theoretical predictions but also to ignite intense research in the field of semiconducting graphene, as our new band-gap engineering protocol for hydrogenated graphene will play a critical role in realizing a broad range of novel applications encompassing integrated carbon electronics, optoelectronics and photonics.

In summary, we have demonstrated that the indirect exposure of CVD graphene to hydrogen plasma successfully modulated the band gap of graphene and, as a result, opened a band gap as wide as ∼4.0 eV without breaking the honeycomb structural symmetry of graphene; we have also shown that our hydrogenated graphene can fully function as a FET at room temperature. All of our hydrogenated graphene with a band gap retained very high structural integrity and was fully reduced to pristine graphene with a zero band gap simply by heating. Our study of monolayer graphene hydrogenated by indirect hydrogen plasma promises to provide answers to many controversial theoretical predictions and to trigger studies on new aspects of semiconducting graphene that have so far been hampered by experimental difficulties. Thus, this new form of graphene should play a significant role in enabling unique applications for long-envisioned carbon-based electronics and photonics.

## Methods

### Preparation of monolayer CVD grapheme

Graphene was prepared under high vacuum by CVD on Cu foils and transferred onto a heavily *p*-doped Si substrate with 300 nm-thick SiO_2_ for Raman, X-ray photoemission spectroscopy, PES and NEXAFS measurements. A sapphire substrate was used and CVD graphene showing ∼2.3% absorption in the visible range was chosen for hydrogenation and optical absorption measurements.

### Hydrogenation

Hydrogen plasma was generated in a separate gun with a microwave power of 180 W at 2.5 GHz. The flow rate of hydrogen was 25 s.c.c.m. and the working pressure was 10 mtorr. The as-prepared CVD graphene was downstream positioned 10 cm away from the end of the gun.

### Device fabrication and electrical measurements

At first, to fabricate graphene-based FETs, electrodes were patterned by a lift-off process after Cr/Pt (5/25 nm) was sputter-deposited on the patterns prepared by photolithography. Then, CVD graphene was transferred onto a heavily *p*-doped Si substrate with 100 nm-thick SiO_2_. PMMA/HSQ bilayer resist on graphene was exposed by an electron beam and HSQ-uncovered PMMA/graphene layers were etched out by O_2_ plasma after developing. After removing them in acetone, FETs with graphene was obtained. They were annealed at 300 °C for 1 h in a gas mixture of Ar/H_2_ (10:1) to remove residual contaminations. Finally, FETs were treated by hydrogen plasma. We measured the electrical properties at various back-gate bias from 2 K to 300 K in vacuum. For a two-probe measurement, we applied constant source-drain voltage (*V*_sd_) and FET mobility was extracted from the differential curve of the electric field effect. For a four-probe Hall measurement, constant source-drain current of 100 nA was applied and the Hall voltage (*V*_H_) was measured. The mobility and carrier density were estimated from *V*_H_ measured by the four-probe Hall measurement.

### Optical band-gap measurements and Raman spectroscopy

The optical transmission and absorption coefficient of graphene and H-Gr were measured at room temperature via visible-ultraviolet spectrophotometry (Cary 5G, Varian Inc.) over the wavelength range of 175–3,300 nm at normal incidence. Raman spectra were obtained with a Horiba Jobin-Yvon Lab Ram HR spectrometer with an Ar laser wavelength of 514.5 nm at room temperature. Diffraction patterns and atomic images were taken by Cs-corrected STEM (JEM-ARM200F, JEOL) with 80 keV electron beam. PES and X-ray photoemission spectra were measured using the synchrotron source at the 4D beam-line and NEXAFS spectra were obtained at the 2 A beamline at Pohang Accelerator Laboratory. The NEXAFS spectra were obtained with partial electron yield at a 55° angle of incidence of the synchrotron photon beam with respect to the surface of the samples.

### Data availability

The data that support the findings of this study are available within the article and its Supplementary Information files, or available from the authors upon request.

## Additional information

**How to cite this article:** Son, J. *et al*. Hydrogenated monolayer graphene with reversible and tunable wide band gap and its field-effect transistor. *Nat. Commun.* **7,** 13261 doi: 10.1038/ncomms13261 (2016).

**Publisher's note:** Springer Nature remains neutral with regard to jurisdictional claims in published maps and institutional affiliations.

## Supplementary Material

Supplementary Information Supplementary Figures 1-4, Supplementary Table 1, Supplementary Notes 1-4 and Supplementary References.
