# Supplementary material for: Hydrogenated monolayer graphene with reversible and tunable wide band gap and its field-effect transistor
Source: Nat Commun. 2016 Nov 10;7:13261. doi: 10.1038/ncomms13261 (PMC5109464; doi:10.1038/ncomms13261)
Supplement: Supplementary Information — Supplementary Figures 1-4, Supplementary Table 1, Supplementary Notes 1-4 and Supplementary References. [file ncomms13261-s1.pdf]

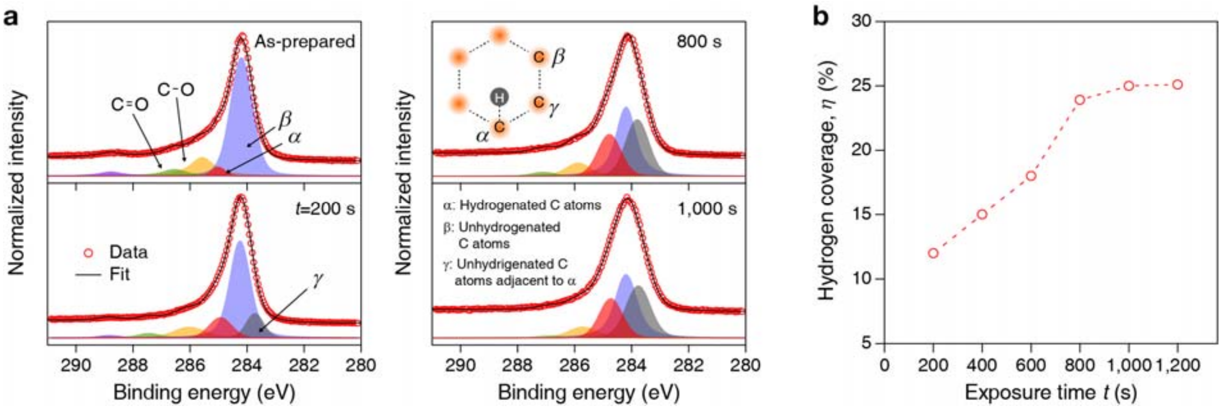

Supplementary Figure 1│ Chemical states of pristine graphene and H-Gr. a, C 1*s* core-level spectra of pristine graphene and H-Gr. The sp^3, sp^2 and H–C–C bonds are denoted as α, β and γ, respectively. **b**, Changes in the hydrogen coverage η with the plasma exposure time *t*.

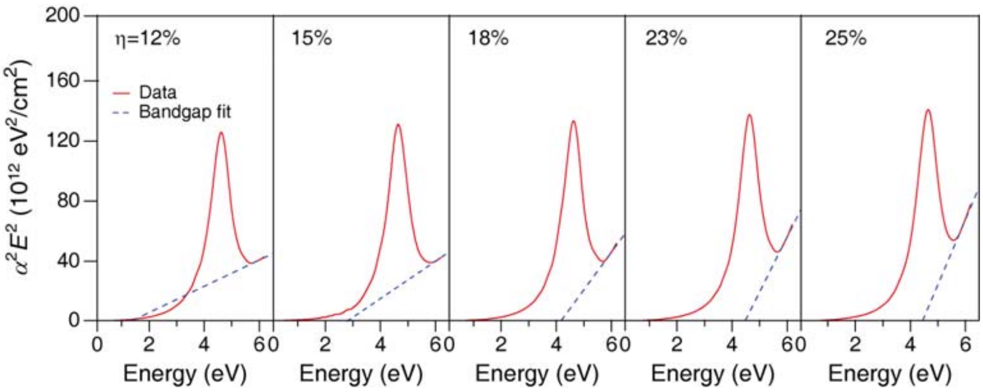

Supplementary Figure 2 | Estimation of an optical bandwidth in H-Gr with various hydrogen coverage (*η*) at 300 K. As *η* increased, the band gap increased and then saturated at ~4.5 eV.

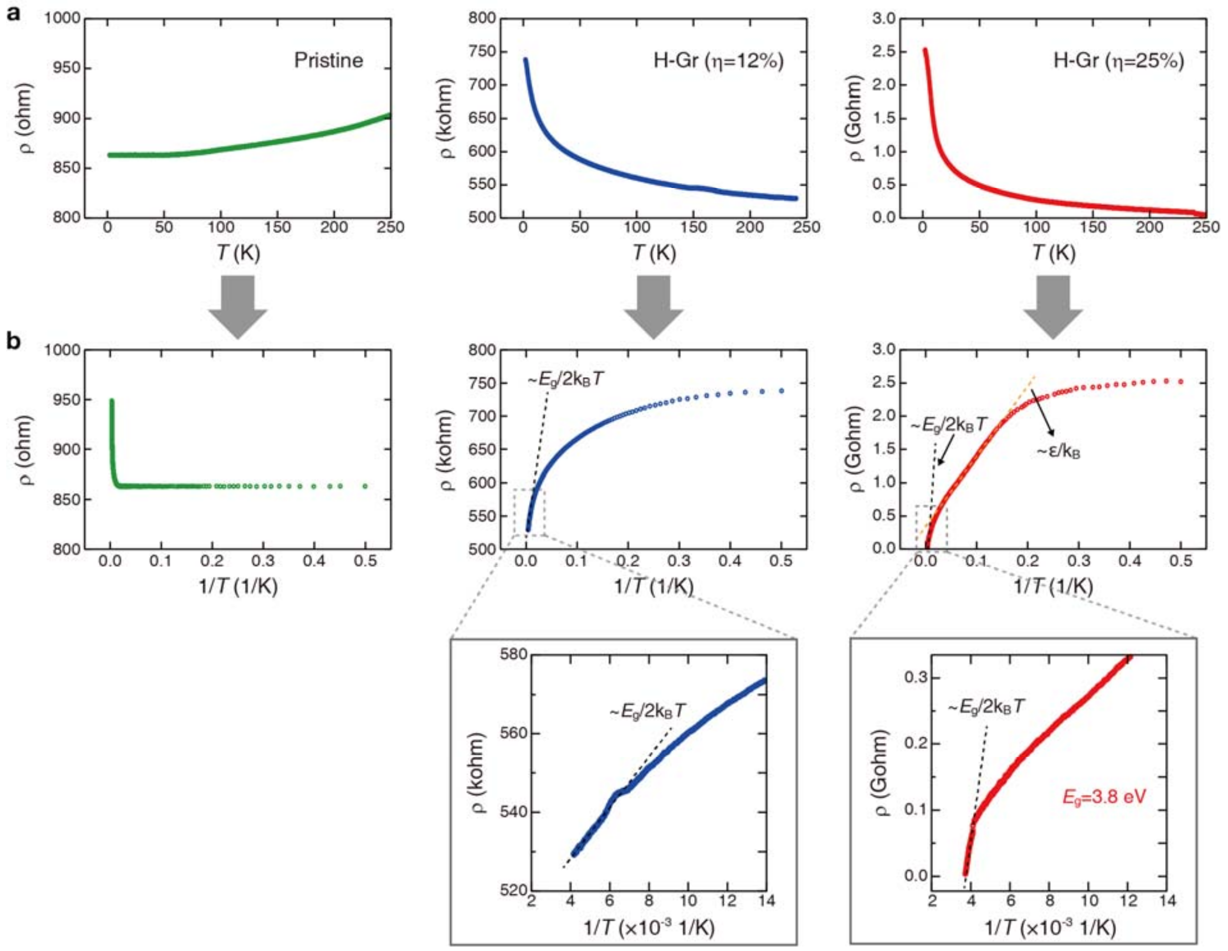

Supplementary Figure 3 │ Temperature dependence of resistivity as evidence of a bandgap in saturated H-Gr. a, ρ–T curves of pristine, H-Gr (η=12%), and H-Gr (η=25%). **b**, ρ–T^{-1} curves of pristine, H-Gr (η=12%), and H-Gr (η =25%).

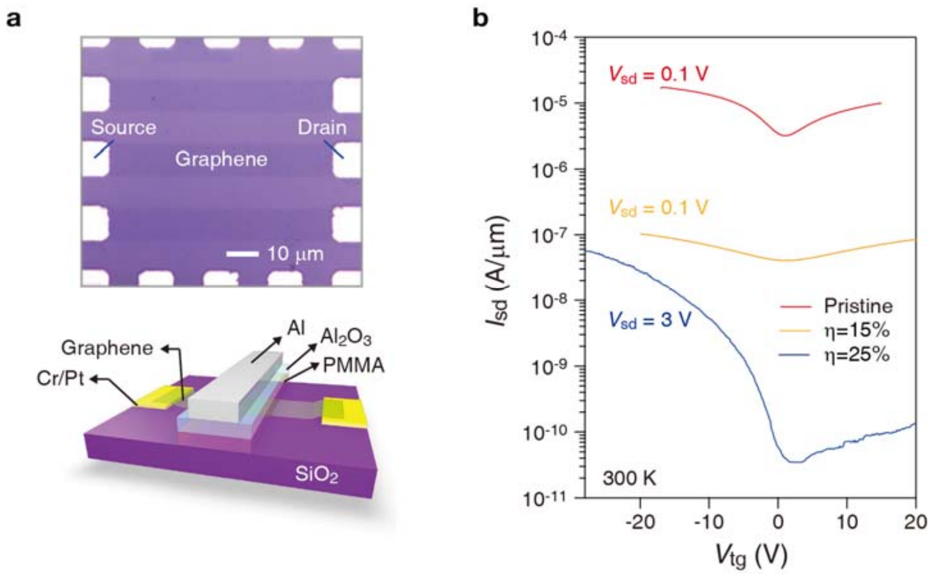

Supplementary Figure 4 | The characteristics of the top-gated field effect transistor (FET) made from H-Gr. a, The optical image and the schematic illustration of the FET; The 60 nm-thick Al-oxide layer and the 50 nm-thick Al electrode were deposited on the 50 nm-thick PMMA layer without breaking vacuum by sputtering. The PMMA/Al-oxide double layer formed a gate dielectric and its capacitance was estimated to be 2.1×10^{-8} Fcm^{-2}. **b**, The *I*–*V* source-drain characteristics as a function of gate bias of the FETs made from pristine and hydrogenated graphene.

Supplementary Table 1│ The electrical properties of pristine graphene and band-gapped H-Gr

		**Pristine graphene**	**H-Gr (η=12%)**	**H-Gr (η=25%)**
R_s (Ω/□)		631.3	107.8×10^3	423.1×10^6
FET measurements	μ (cm^2/V·s)	2,555.6	100.7	2.8
Hall measurements (at V_{bg}=0 V)	μ (cm^2/V·s)	5,229.4	273.6	3.7
	Carrier concentration (cm^{-2})	6.8×10^{12}	4.5×10^{12}	3.8×10^{11}

Supplementary Note 1

Estimation of hydrogen coverage by XPS.

We found that in the case of a band gap of 3.9 eV, about 25% was the hydrogen coverage η as calculated by quantitative analyses of the C 1*s* core-level measured by synchrotron radiation X-ray photoelectron spectroscopy (XPS), which corresponds to stoichiometric C_4H[1,2]. In the calculation, we assumed that hydrogen atoms are chemically bonded to carbon atoms on the top surface only since the surface of graphene on a SiO_2/Si substrate is indirectly exposed to the plasma. The C 1*s* spectrum contains various bonding types, such as sp^3, sp^2 and H–C–C, of hydrogenated graphene, which we call α, β and γ, respectively. The CVD graphene is mainly composed of β and also has oxidic phases (Supplementary Figure 1a). As we increased the exposure time *t*, the α and γ peaks gradually became stronger while the β peak became weaker, which indicates that hydrogen is covalently bonded with carbon in graphene. The relative portion of both α and γ peaks started to saturate over an exposure time of 800 s, corresponding to an η of 25%, at which the optical bandwidth was also maximally saturated (Supplementary Figure 1b). The saturation implies that hydrogen in excess did not react further with carbon when η reached 25%. The H-Gr should therefore be a stoichiometric form of C_4H.

Supplementary Note 2

Estimation of a higher-lying direct bandwidth in H-Gr by optical absorption.

The optical transmission T of graphene and H-Gr on a sapphire substrate was measured in the visible-ultraviolet (VUV) region by grating spectrophotometry (Supplementary Figure 2). With the transmission normalized against the substrate effect, the optical absorption coefficient α was obtained according to the standard formula

$$\alpha = -\frac{1}{d}\ln T \,, \qquad \text{Eq. 1}$$

where d is the thickness of the sample. In general, the absorption coefficient due to an interband transition of photo-excited electrons is described by

$$\alpha(\nu)h\nu = B\left(h\nu - E_g\right)^{\beta} \,, \qquad \text{Eq. 2}$$

where B is a material-dependent constant, $h\nu$ is the incident photon energy, and E_g is the threshold energy for interband absorption (often a band gap itself for a direct band-gap semiconductor). Our higher-lying absorption background is best fitted with $\beta\approx 1/2$, i.e., a direct interband transition type. The E_g for our H-Gr is determined by the energy-intercept of the line tangent to the high-energy (4-6 eV) absorption tail of α^2E^2.

Supplementary Note 3

Temperature dependence of resistivity of pristine and hydrogenated graphene.

We search for gapped-semiconductor behavior from our ρ–T data of three types of graphene films (pristine, H-Gr (η=12%), and H-Gr (η=25%)) over the temperature range of 1.8-250 K. As shown in Supplementary Figure 3a, the resistivity (ρ) of pristine graphene monotonically decreases with decreasing temperature, revealing its metallic transport characteristics, which include saturation behavior toward zero temperature. In sharp contrast, both H-Gr films exhibit insulator-like transport characteristics as their resistivity increases with decreasing temperature. While the resistivity of H-Gr (η=12%) can be fitted well with a single power-law exponent close to -1/3, suggesting variable-range hopping (VRH), over the entire temperature range of our measurement, H-Gr (η=25%) exhibits three distinct regions characterized by (1) an exponential dependence, consistent with the intrinsic region with a gap of 3.8 eV; (2) a power-law type ($T^{-1/3}$), consistent with the VRH regime; and (3) saturation behavior, possibly headed toward the Mott minimum conductivity. If we force the gapped-model fit to the resistivity of H-Gr (η=12%) within a narrow interval of the high temperature region, we obtain a gap energy of 0.02 eV, which deviates substantially from the bandgap (~2 eV) of H-Gr (η=12%) estimated by synchrotron radiation spectroscopy or optical absorption experiments. This result means that H-Gr (η =12%), with a hydrogen concentration level lower than 25%, could actually have been driven to be a disordered state by random hydrogenation. In contrast, for H-Gr (η=25%, E_g~3.9 eV), the ρ–T^{1} fit in the high temperature region clearly shows evidence of an intrinsic band gap of ~3.8 eV, which is quite consistent with that (~3.9 eV) obtained by synchrotron radiation spectroscopy or optical absorption experiments.

Supplementary Note 4

Carrier mobility and concentration of pristine and hydrogenated graphene.

In order to obtain FET mobility by 2-probe measurement, we applied constant source-drain voltage (V_{sd}), and FET mobility (μ) was extracted from the differential curve of the electric field effect according to the standard formula

$$\mu = \frac{L}{W \cdot C_i \cdot V_{sd}} \left(\frac{\Delta I_{sd}}{\Delta V_{bg}} \right), \qquad \text{Eq. 3}$$

where L (W) is length (width) of graphene channel. C_i is the gate capacitance and it was 3.0×10^{-8} F/cm^2 in our 100-nm-thick SiO_2. V_{bg} is the back gate bias voltage. I_{sd} is the source-drain current measured by the electric field effect measurement.

For 4-probe Hall measurement, constant I_{sd} of 100 nA was flowed, and Hall voltage (V_H) was measured. The mobility and carrier concentration (n) were calculated according to the standard equation of Hall measurement,

$$\mu = \frac{V_H \sigma}{I \cdot B} = -\frac{\sigma}{ne}, \qquad \text{Eq. 4}$$

where B is the external magnetic field, σ the conductivity of graphene and e the elementary charge.

Supplementary References

1. Li, Y. & Chen, Z. Patterned partially hydrogenated graphene (C_4H) and its one-dimensional analogues: A computational study. *J. Phys. Chem. C* **116**, 4526-4534 (2012).
2. Ma, Y. *et al.* Electronic and magnetic properties of the two-dimensional C_4H-type polymer with strain effects, intrinsic defects and foreign atom substitutions. *Phys. Chem. Chem. Phys.* **14**, 3651-3658 (2012).
